# Factors Influencing Contrast Enhancement in Abdominal Computed Tomography Angiography in the Dog: A Systematic Review

**DOI:** 10.3390/ani14233521

**Published:** 2024-12-05

**Authors:** Simone Perfetti, Carlo Guglielmini, Nikolina Linta, Alessia Diana

**Affiliations:** 1 Department of Veterinary Medical Sciences, University of Bologna, 40064 Ozzano dell’Emilia, Italy; simone.perfetti4@unibo.it (S.P.); nikolina.linta2@unibo.it (N.L.); alessia.diana@unibo.it (A.D.); 2 Department of Animal Medicine, Production and Health, University of Padua, 35020 Legnaro, Italy

**Keywords:** abdominal disorders, abdominal vascularization, canine, diagnostic imaging, contrast medium

## Abstract

Multidetector-row computed tomographic angiography (angio-CT) aims to achieve optimal opacification of the vascular compartment of interest. The distribution and quality of vascular opacification are influenced by patient-related factors, contrast medium (CM)-related factors, and scanner-related factors. This systematic review evaluates these factors and their effects on contrast enhancement. A comprehensive literature search was made in February 2024 across four online bibliographic databases (Web of Science, PubMed, Scopus, and CAB Abstract) in adherence with the PRISMA 2020 guidelines. After screening the 5990 unique published articles initially identified, 20 full-text original studies met the inclusion criteria for the final review. The amount of abdominal adipose tissue was found to significantly affect enhancement, which suggests the possibility of reducing the CM dose to minimize adverse effects or toxicity. The injection rate of the CM, rather than the injection duration, was identified as the most critical factor, with important clinical implications. For scanners with slower acquisition speeds or longer scan durations, maintaining a fixed CM injection duration may optimize vascular phase acquisition. In contrast, faster scanners benefit from bolus tracking, which allows for improved differentiation between vascular phases. Additionally, administering a saline flush post-CM injection enhances arterial opacification while reducing the necessary CM dose. This systematic review highlights essential factors influencing contrast enhancement in angio-CT for dogs and provides a foundation for future research aimed at optimizing imaging protocols in veterinary medicine.

## Simple Summary

Computed tomography (CT) is the preferred imaging modality for evaluating parenchymal lesions and vascular disease in dogs. In recent years, numerous studies have investigated the factors influencing contrast enhancement in canine abdominal CT studies. In this study, we aimed to identify the key factors affecting enhancement during abdominal CT in dogs, conducting a comprehensive analysis and critical evaluation of the veterinary literature according to the PRISMA 2020 guidelines. Among the patient-related factors, the amount of abdominal adipose tissue emerged as the most significant, suggesting that the contrast medium (CM) dose could potentially be adjusted based on the patient’s lean body weight. The injection rate of the CM was identified as the most influential CM-related factor, particularly at the arterial and hepatic levels, with potential clinical implications. Furthermore, the administration of a saline flush following CM injection was shown to improve arterial enhancement while reducing the overall CM dose.

## 1. Introduction

Multidetector-row computed tomography angiography (angio-CT) is the imaging technique of choice for assessing various vascular diseases, including portosystemic shunts, arteriovenous fistulas, thromboembolism, and neoplastic vascular invasion [1,2,3,4]. Angio-CT allows for the differentiation of vascular phases by capturing arterial, portal, and venous phases, providing detailed insights into the vascular system. A key aspect of this technique is the use of a contrast medium (CM), typically an iodinated agent, which enhances imaging by distributing through two distinct phases: the vascular phase, where the CM remains in the bloodstream, and the interstitial phase, where the CM moves from the bloodstream into the surrounding interstitial compartments [4,5,6,7].

The primary objective of angio-CT is to achieve optimal opacification of the vascular compartments of interest (referred to as “enhancement”) and synchronize this process with the CT scan, a task that has become increasingly complex with advancements in CT scanner technology. The distribution of contrast medium (CM) and the resultant opacification of critical anatomical regions—whether vascular or parenchymal—are influenced by a variety of factors, including patient characteristics, CM properties, and scanner parameters. In human medicine, these influencing factors are well understood and have been extensively studied, with the aim of minimizing both radiation exposure and the volume of CM administered [6,7]. However, in veterinary medicine, particularly in dogs, these factors remain under-researched, despite the growing application of CT in routine clinical practice. One of the key uses of angio-CT in veterinary medicine is the evaluation of the abdominal cavity, where it is employed to diagnose a wide range of conditions, from congenital or acquired vascular abnormalities to various neoplastic diseases [1,2,4].

The aim of this study was to conduct a systematic review of the existing literature on the factors influencing the enhancement of major abdominal organs and vascular structures during angio-CT in dogs. Additionally, we compared the findings of the canine literature on this topic with those reported in human studies. 

## 2. Materials and Methods

### 2.1. Literature Search Strategy 

For this study, a systematic review of peer-reviewed literature was conducted to identify studies evaluating the factors influencing vascular or parenchymal enhancement in abdominal angio-CT in dogs. The review adhered to the Preferred Reporting Items for Systematic Reviews and Meta-Analyses (PRISMA) 2020 guidelines. A single reviewer performed the literature search using multiple online databases, including PubMed, Web of Science, Scopus, and CAB Abstracts, covering studies from the inception of each database through February 2024. The search strategy employed specific keywords combined using Boolean operators such as “AND” and “OR”. Table 1 summarizes the keywords used in this search. All studies identified through the review were organized using bibliographic reference management software (Zotero, version 6.0.37). For each study, key details such as the authors’ names, publication year, journal title, volume, issue, and page numbers were recorded.

### 2.2. Study Selection

After collecting the studies, duplicates were removed from the database. Two authors (SP and NL) independently reviewed the titles and abstracts to exclude non-veterinary studies, except those involving dogs in experimental evaluations of CT protocols. The relevance of each study to the inclusion criteria was assessed, and the reference lists of all selected papers were checked for additional citations not captured in the initial search. Any new citations were screened for inclusion in the final corpus. In cases of disagreement, a consensus on which articles to include for full-text review was reached through discussion, with a third author (AD) consulted when necessary. Following this, two researchers (SP and NL) independently evaluated the full texts to confirm eligibility. Any discrepancies were again resolved through discussion, with the option of involving a third-party adjudicator (AD) if required.

### 2.3. Data Extraction

One author (SP) extracted data from the eligible studies. The descriptive variables collected included the author’s name, publication year, study design, category of factors evaluated, and sample size.

### 2.4. Inclusion and Exclusion Criteria

In our review, we included studies published in peer-reviewed journals without any temporal limitations. The studies had to be written or translated into English, with no restrictions on the authors’ nationality. Each study was required to meet the following inclusion criteria to be considered for the systematic review:

- Peer-reviewed articles published in English that evaluated factors influencing enhancement in canine angio-CT.
- Articles reporting primary research results, including case series, observational cohort studies, cross-sectional studies, case–control studies, and randomized controlled trials; literature reviews and single case reports were excluded.
- Full-text papers; studies for which only an abstract was available (i.e., congress proceedings) were excluded.
- Clinical or experimental studies utilizing a canine model that included clinically healthy dogs.

### 2.5. Quality Assessment

To assess the quality of the eligible full-text articles, we utilized the Evidence Quality Grading System tool developed in 2013 by the National Heart, Lung, and Blood Institute (NHLBI) of the National Institutes of Health (NIH) (available at https://www.nhlbi.nih.gov/health-topics/study-quality-assessment-tools, accessed on 23 October 2023). Two independent reviewers (SP and NL) conducted the quality assessments, resolving any discrepancies through consultation with a third reviewer (AD). Using this tool, the strength of evidence from various types of study (i.e., observational cohort studies, cross-sectional studies, and case series) is evaluated across a set of 12 or more questions. Three responses are possible for each question: Yes, No, or Other (i.e., cannot determine, not applicable, or not reported). Accordingly, the criteria to classify the selected studies were as follows:

High-quality studies (Good): Yes for all criteria.

Moderate-quality studies (Fair): Yes for most criteria.

Low-quality studies (Poor): No or Other for most criteria.

## 3. Results

**Figure 1:**
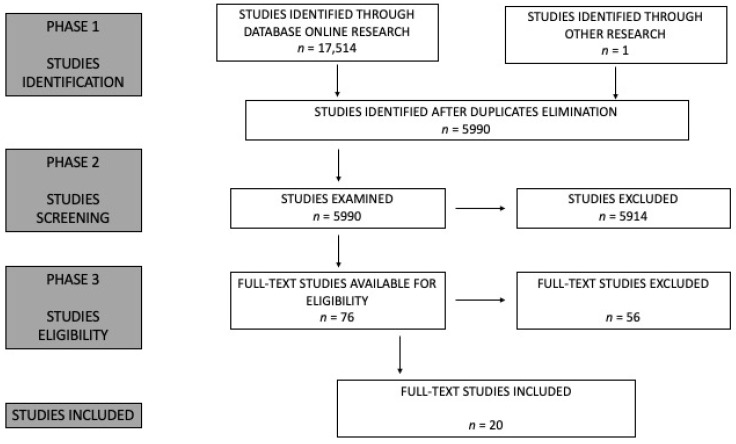
Flow diagram of the literature search strategy.

The bibliographic search identified 17,514 studies, which were collected using the reference management software Zotero. After removing duplicates, 5990 studies were analyzed for subsequent selection based on titles and abstracts, resulting in 76 studies being identified. Following a full-text evaluation, 20 studies met all the inclusion and exclusion criteria and were included in the final corpus of the systematic review. A schematic representation of the analysis is shown in Figure 1, and the list of included studies is provided in Table 2. Notably, 19 of the 20 studies were identified through online database searches, while one study was located by reviewing the bibliographic references of eligible studies. 

In the final corpus, no studies were classified as high quality. Nine articles (45%) were found to have moderate reporting quality [8,9,10,11,12,13,14,15,16], while 11 articles (55%) were rated as having low reporting quality [17,18,19,20,21,22,23,24,25,26,27]. The most common reasons for lower quality ratings included a lack of justification for sample size and methodological inadequacies.

**Table 2:** Summary details of the 20 studies included within the final systematic review evaluating factors associated with enhancement in abdominal computed tomographic angiography in the dog.

First Author	Year	Study Design	Factor’s Category	Number of Dogs	Quality Rating
Tateishi K. [20]	2008	PC-O	CM	5	Poor
Kan J. [17]	2021	RC-S	Patient	62	Poor
Kim C. [16]	2020	PC-O	CM	6	Fair
Burgener F.A. [22]	1981	Prospective	CM	10	Poor
Lee S.K. [14]	2017	PC-O	CM	6	Fair
Kishimoto M. [11]	2008	PC-O	CM	4	Fair
Amaha T. [19]	2022	Prospective	CM	5	Poor
Blaser A. [12]	2016	PC-O	CM	9	Fair
Kutara K. [18]	2020	PC-O	Patient	5	Poor
Choi S.Y. [21]	2018	Prospective	CM	8	Poor
Choi S.Y. [24]	2015	Prospective	Scanner	6	Poor
Chau J. [13]	2016	Prospective	CM	18	Fair
Davè A.C. [10]	2022	Retrospective	Patient, CM, Scanner	233	Fair
Fitzgeral E. [15]	2017	RC-S	CM	35	Fair
Siow J.W. [27]	2023	RP	Scanner	70	Poor
Kan J. [8]	2022	PC-S	Patient	12	Fair
Choi S.Y. [25]	2016	PC-O	Scanner, CM	8	Poor
Cho H. [26]	2018	Prospective	Scanner	6	Fair
Kim H. [23]	2019	PC-O	CM	5	Poor
Thierry F. [9]	2018	RP	Scanner, CM	51	Fair

Abbreviations: CM = contrast medium; PC-O = prospective cross-over; PC-S = prospective cross-sectional; RC-S = retrospective cross-sectional; RP = retrospective–prospective.

No temporal limitations were imposed on the studies included in our systematic review, which spanned from 1981 to 2022. The majority of the studies (16 out of 20, or 80%) were published from 2016 onwards [8,9,10,12,13,14,15,16,17,18,19,21,23,24,25,26,27]. In terms of study design, eight studies were classified as prospective cross-over (40%) [11,12,14,16,17,18,23,24], six as prospective (30%) [13,19,21,22,25,26], two each as retrospective cross-sectional [15,17] and mixed retrospective–prospective [9,27] (10%), and one each as retrospective [10] and prospective cross-sectional [8] (5%). The median study population consisted of 8 dogs, with a range of 5 to 233 animals. Retrospective and mixed retrospective–prospective studies featured larger populations, with a median of 62 dogs (range: 51 to 233).

Regarding the factors related to enhancement, most studies (14 out of 20, or 70%) focused on contrast medium (CM)-related factors [9,10,11,12,13,14,15,16,19,20,21,22,23,24], six studies (30%) examined CT-related factors [9,10,24,25,26,27], and four studies (20%) investigated patient-related factors [8,10,17,18]. One study (5%) evaluated all three categories of factors simultaneously [10]. Of the 14 studies on CM-related factors, 11 (82%) concentrated on the influence of the injection protocol (in terms of technique or speed) on enhancement [10,11,12,13,15,16,19,20,22,24]. Additionally, one study (9%) examined the composition of the CM and the effect of a saline flush at the end of CM administration [22,23]. Among the five studies on scanner-related factors [10,11,24,25,26,27], four (80%) investigated scan delay to optimize enhancement of specific targets [10,24,25,26,27], while one study assessed the effect of the type of scanner on enhancement [11]. For patient-related factors, two studies (50%) explored the influence of abdominal adipose tissue [8,17], and one study (25%) investigated the anesthetic protocol used and the patient’s heart rate [18].

### 3.1. Patient-Related Factors

Comprehensive results summarizing the identified patient-related factors for each study in this review are presented in Table 3. The most commonly studied factors included body weight (specifically abdominal fat percentage), heart rate, and the anesthetic protocol.

For body weight, a significant relationship was found between the percentage of abdominal adipose tissue and enhancement in the aorta, liver, and portal vein during the portal phase. Body weight appeared to be positively associated with a delayed venous phase in some abdominal organs, such as the liver and kidneys, although it did not significantly affect overall image quality.

Heart rate was identified as the most influential factor impacting the accuracy and timing of the vascular phases. One study reported differences in vascular and hepatic enhancement between dogs under general anesthesia (induction with propofol and maintenance with isoflurane) and those under sedation (intramuscular administration of medetomidine). Specifically, the arrival of the contrast medium and the peak enhancement in the aorta, portal vein, and liver parenchyma were significantly delayed in sedated dogs compared to those under general anesthesia, with delays of up to 20 s.

### 3.2. Contrast Medium-Related Factors

Comprehensive results summarizing the identified CM-related factors for each paper in this review are presented in Table 4. One study evaluated the effect of iodine concentration, finding that the use of a high iodine concentration CM (i.e., 350 mgI/mL) did not result in greater enhancement compared to formulations with lower concentrations (i.e., 180–320 mgI/mL). Additionally, the amount of CM injected did not influence the time–density curve along the temporal axis; it only determined a higher peak of enhancement in the aorta, portal vein, and liver.

The injection technique was evaluated in 11 studies, focusing on the injection speed of the CM, the injection rate (specifically, the speed of iodine transport), and the duration of the injection. The duration and technique of injection significantly influence the aortic peak of enhancement, accounting for 96% of the variables affecting the time to peak aortic enhancement. In contrast, the duration of the injection does not significantly impact hepatic and portal enhancement. Similarly, injection duration and technique do not affect the time to peak enhancement of the adrenal glands.

Regarding the injection technique, studies indicated that bolus testing and bolus tracking yield better quality arterial studies compared to fixed injection duration protocols. Conversely, fixed injection duration is more suitable for the portal phase. Specifically, bolus testing is more effective with slow scanners, while bolus tracking performs better with high-performance scanners. The fixed injection duration technique can be utilized with both slow and fast scanners, making it easily reproducible.

The injection technique for gastrointestinal enhancement was evaluated in two studies using a dual-injection method. This technique allows for greater enhancement of the intestinal wall compared to the classic single injection technique, which provides adequate enhancement of mesenteric vascularization. However, this advantage does not apply to the duodenum and ileum, which are characterized by blurry images.

The influence of catheter size on angio-CT enhancement was studied in one paper; an injection rate of 1.5 mL/s using a 24 G catheter resulted in adequate aortic and portal enhancement peaks, with good differentiation of the various vascular phases. Additionally, one study assessed the use of a saline flush at the end of contrast medium administration. This study found no significant reduction in vascular enhancement (particularly arterial and portal) or hepatic enhancement after reducing the dose of contrast medium by approximately 30% compared to the minimum recommended dose (600 mgI/kg). Furthermore, using a flush following the contrast medium bolus increased peak arterial enhancement but did not affect portal or hepatic enhancement.

### 3.3. Scanner CT-Related Factors 

Comprehensive results summarizing the identified scanner-related factors for each paper in this review are presented in Table 5. Most studies (4 out of 6, or 66%) aimed to evaluate and determine the optimal scanning delay based on the administration of the CM bolus. Some studies proposed predefined delays tailored to specific target organs, including the pancreas, stomach, small intestine, and kidneys.

One study examined both the type of injection protocol and the influence of the CT scanner type on vascular enhancement during angio-CT. Notably, it was observed that the quality of the arterial phase, in terms of enhancement, is significantly higher when using a 64-slice CT scanner compared to less advanced scanners (4–16 slices). Additionally, the length of the scan can influence vascular and parenchymal enhancement; a multifactorial study found that liver and kidney enhancement is particularly affected by scan duration.

## 4. Discussion

The results of this systematic review highlighted factors influencing vascular and parenchymal enhancement during abdominal angio-CT studies in dogs, categorized into three major groups: patient-related factors, CM-related factors, and scanner (or scanning technique)-related factors. This differentiation is consistent with descriptions in human medicine [6]. Factors from different categories can influence one another and are often studied concurrently. When evaluating multifactorial studies—specifically those that encompass factors from various categories—we focused particularly on the factor most thoroughly covered in each study.

Regarding the evaluation of patient-related factors, there is a small number of studies in veterinary literature, which is surprising given the significant intra-species variability among dogs. In both human and canine literature, the patient-related factors that most influence enhancement during CT angiography are cardiac output and body weight [4,6]. In dogs, the most extensively studied factor is the amount of body adipose tissue [8,17]. A significant relationship has been observed between the percentage of abdominal adipose tissue and enhancement at the level of the aorta, liver, and portal vein during the portal phase. This association is likely due to a relative increase in the iodine dose administered in relation to lean body weight in dogs with moderate to severe abdominal fat [17].

In most cases, varying the CM dose based on lean body weight results in reduced enhancement compared to the dose calculated based on total body weight, without an apparent decrease in the diagnostic quality of the CT examination [8]. For this reason, it is recommended that the dose of injected CM be calculated based on lean body weight rather than total body weight. This approach would particularly benefit obese animals, similar to humans, by reducing the total dose of CM administered and consequently minimizing potential adverse effects, such as CM-induced nephropathy [28].

Furthermore, body weight appears to be positively associated with a delayed venous phase in some abdominal organs, such as the liver and kidneys, although it does not seem to influence overall image quality [10]. The relationship between body weight and renal and hepatic venous enhancement is complex and likely linked to multiple factors, including the patient’s body weight and size, which can influence the length and duration of the scan [10,11].

Cardiac output is another important factor influencing enhancement in angio-CT examinations. Heart rate directly affects flow rate, which is the most significant determinant of the timing of enhancement. Specifically, a reduction in cardiac output leads to a delay in the arrival of the CM and the peak enhancement [4,6]. Additionally, cardiac output is influenced by the use of various anesthetic drugs [29]. This consideration is particularly relevant in veterinary medicine, as CT examinations are typically performed on animals under general anesthesia or sedation.

The administration of a highly selective α2-agonist, such as medetomidine, induces vasoconstriction and bradycardia, which subsequently affects the distribution of the CM [29]. Therefore, it is advisable to adjust the scan times compared to those commonly used for animals under general anesthesia, specifically by increasing the scan start delay when performing angio-CT studies on dogs sedated with medetomidine. The anesthetic protocol is a significant factor influencing angio-CT enhancement in dogs—a consideration that is not commonly evaluated in humans due to the infrequent use of anesthesia during CT examinations.

Other patient-related variables, such as systolic, diastolic, and mean arterial pressure, do not appear to significantly influence the timing of enhancement in dogs, which contrasts with findings in humans [11].

The main CM-related factors influencing vascular enhancement include the type and volume of CM administered, its concentration and pharmacokinetics, the duration and technique of injection, and the use of a saline flush [6,7]. CM-related factors were the most frequently evaluated in the included studies, revealing significant variability in CM administration techniques among different institutions due to a lack of standardized protocols for dogs. Proper CM administration has important clinical implications, particularly concerning toxicity and potential adverse effects associated with CM itself. For instance, the onset of renal damage and adverse reactions such as bradycardia, tachycardia, and hypotension or hypertension have been reported in dogs and cats [30,31].

Interestingly, using a high iodine concentration CM does not necessarily result in greater enhancement compared to formulations with lower concentrations. This phenomenon may be linked to a higher concentration of residual CM at the injection site in higher concentration formulations, which tend to have a greater total volume and, consequently, a lower effective dose of CM at the regional target level [11]. Furthermore, at the same administered dose, low-concentration formulations can lead to a significant reduction in enhancement compared to those with higher concentrations, likely due to a greater dilution of iodine relative to the total administered volume [11]. It is also important to note that low-concentration formulations have a shorter arrival time of the CM due to their lower viscosity [11]. The amount of CM also significantly influences hepatic and adrenal perfusion [12,19].

Most articles included in this review that evaluated CM-related factors focused on injection technique and speed. These issues are easier to assess and are of great clinical interest, particularly regarding the appropriate dose of contrast medium to inject. A constant injection rate helps reduce CM dispersion and achieves a shorter time to peak aortic and/or hepatic enhancement compared to constant rate administration [13,20]. During abdominal angio-CT, the regression formula (0.8 × [duration of the injection + arrival time of the CM] ± constant) aids in estimating the peak time of aortic enhancement and calculating the necessary scanning delay [13].

However, the duration of the injection is not a significant factor for hepatic and portal enhancement; nonetheless, extending the injection duration may lead to greater persistence of enhancement at the hepatic level, though this finding is not statistically significant [13,19,20,21]. In humans, the ideal injection rate for obtaining an adequate multiphasic study with a high-quality arterial phase is 3 mL/s, which requires a venous catheter of at least 22 G [6]. The use of large-caliber venous catheters can be a limitation in small animals, such as cats and toy-breed dogs. Studies have demonstrated that an injection rate of 1.5 mL/s provides adequate aortic and portal enhancement peaks, with good distinction among the various vascular phases [14]. This finding applies to CM injection durations of up to 15 s. When the injection rate is less than 15 s, the peak enhancement time is primarily determined by the arrival time of the CM at the target organ level, with minimal influence from the injection duration [14]. Therefore, in small animals requiring smaller catheters (e.g., 24 G), it is still possible to achieve a triphasic angio-CT study characterized by good distinction among the vascular phases and high-quality diagnostic images.

In both dogs and humans, techniques such as bolus testing and bolus tracking yield better quality arterial studies compared to fixed injection duration protocols [6,10]. However, the opposite is true for the portal phase; specifically, bolus testing is more suitable for slower scanners, while bolus tracking is preferable for high-performance scanners. The fixed injection duration technique can be used with both slow and fast scanners, making it easily reproducible [10]. In dogs, a dual-injection technique may be beneficial for animals with gastrointestinal disorders (e.g., ischemia or occlusion) when the length or duration of the scan does not allow for the acquisition of both the arterial and portal phases [15]. This method involves fewer images to interpret than the biphasic modality and results in less radiation exposure for the patient. Furthermore, this approach provides greater enhancement of the intestinal wall compared to the classic scanning technique, with the exception of the duodenum and ileum, which are characterized by blurry images [15].

In humans, the effects of using a saline flush at the end of CM administration on CT enhancement have been widely described. This technique helps increase the attenuation peak and reduce CM artifacts by minimizing the total amount used [6,7]. In contrast, our systematic review identified only one study evaluating the effect of a saline flush on the CM dose in dogs, which resulted in increased peak arterial enhancement without a significant reduction in vascular and parenchymal enhancement [23]. The clinical application of this technique could be particularly beneficial for animals with renal dysfunction, where the administration of large amounts of iodinated CM is not recommended.

The evaluation of scanner-related factors is relatively underexplored in dogs, likely due to the recent availability of high-performance scanners, which has limited the number of studies in this area. Key parameters influencing enhancement include the duration and direction of the scan, the multiphasic acquisition during different enhancement phases, the definition of the arrival time of the CM concerning the scan delay, and the timing of the scan following CM administration [4,6].

Notably, the quality of arterial phase enhancement is significantly higher with 64-slice CT scanners compared to less advanced scanners with 4–16 slices; however, this superiority has not been demonstrated for the portal phase [10]. The number of detectors impacts scanning speed, allowing for high-quality images across all vascular phases, regardless of the injection protocol employed. In contrast, slower scans conducted with less advanced scanners exhibit greater variability in enhancement quality across different vascular phases [10].

Most veterinary studies have concentrated on evaluating and determining the optimal scanning delay based on the administration of the CM bolus. Some studies have proposed predefined delays tailored to specific target organs, such as the pancreas, stomach, small intestine, and kidneys [24,25,26,27]. Establishing these delays can help mitigate the effects of inter-individual variability concerning CM transit time, enable lower CM doses, and reduce radiation exposure [26]. However, angio-CT examinations in small animals often cover the entire abdominal or thoracoabdominal area, typically employing standardized protocols for the entire region rather than for individual organs. When a specific organ is of primary interest, it may be beneficial to adjust the scanning delay accordingly, without significantly affecting the overall evaluation of the region examined. Furthermore, it is advisable to modify the scan length to ensure the minimum necessary duration for assessing the organs of interest.

In clinical practice, thoracic scans are frequently included alongside abdominal scans, resulting in significantly increased scan lengths and durations. Therefore, thoracic scans should only be performed during abdominal CT angiography when essential for diagnostic purposes (e.g., oncological staging) or conducted as a final scan to avoid compromising the enhancement of areas of interest.

The studies included in this systematic review exhibited considerable variability in study design, sample size, and methodology, all of which must be considered when interpreting the findings. Notably, the lack of standardization in the protocols used impacted the evaluation of the various factors examined. This systematic review encompasses studies published from 1981 to 2022. However, with the exception of one study from 1981 [22], the remaining articles primarily fall within a narrower timeframe from 2008 to 2024, which limits variability in the methodologies and technologies employed.

The rather limited number of available studies underscores the need for further exploration of this topic in the coming years, particularly given the increasing use of this technique in veterinary clinical practice. No selection was made based on study design, recognizing the limitations of retrospective studies in assessing the variability of the examined factors. Experimental studies involving canines were also included to broaden the range of articles considered. Although the meticulous selection process aimed to minimize human error, the potential for such errors in the number of included items still exists. The review of the references of included articles, which facilitated the incorporation of previously unidentified studies, serves as a valuable strategy to mitigate potential mistakes during initial screenings, although it does not entirely eliminate the risk of human error. Furthermore, it is important to note that none of the studies reviewed achieved a high-quality classification, and half were classified as poor quality. This indicates that the overall quality of veterinary studies in this area remains inadequate. Such limitations emphasize the necessity for a nuanced interpretation of the findings from this systematic review. While the identified factors influencing angio-CT enhancement offer valuable insights, the overall quality ratings of the studies should temper the confidence with which these factors are applied in clinical practice.

The heterogeneity of literature is demonstrated by the notable variability of topics addressed in the included studies. This lack of consistency impedes the establishment of standardized protocols for abdominal CT angiography in veterinary medicine. Defining such protocols would enhance the repeatability and reproducibility of CT techniques, promote multicenter studies with larger animal populations, and ultimately improve the overall scientific quality of the field.

## 5. Conclusions

In summary, this systematic review has underscored the factors influencing vascular and parenchymal enhancement during abdominal angio-CT in dogs. Notably, the quantity of abdominal adipose tissue is an intriguing yet often overlooked factor in daily clinical practice. Recognizing this factor may facilitate a reduction in CM dosage, thereby decreasing the risk of adverse effects and toxicity.

The speed of iodine transport emerges as more significant than the duration of injection, with important clinical implications, particularly in the evaluation of hepatocellular carcinomas. A fixed duration for CM injection may be advisable when using slower scanners or during lengthy scans, while the bolus-tracking technique provides improved enhancement quality across various vascular phases with more advanced scanners.

Lastly, implementing a saline flush at the end of CM administration is recommended to enhance arterial enhancement and may allow for a reduction in the CM dose.

## Figures and Tables

**Table 1:** Keywords used in the systematic review: factors affecting enhancement in abdominal angio-CT in dogs.

Species	AND	Machine	AND	Key Word
Dog		CT		Protocol
OR		OR		OR
Canine		Computed tomography		Standard
				OR
				Abdomen
				OR
				Vascular
				OR
				Factors

**Table 3:** Summary details of the study included in the final systematic review evaluating patient-related factors associated with an enhancement in abdominal angio-CT in the dog.

First Author	Year	Patient-Related Factor	Anatomical District
Kan J. [17]	2021	Lean body weight/abdominal fat percentage	Ao, PV, liver, kidney, spleen
Kutara K. [18]	2020	Anesthetic protocol	Ao, CVC, PV, liver
Davè A.C. [10]	2022	Heart rate	Ao, CVC, PV, liver, pancreas, spleen, kidney
Kan J. [8]	2022	Lean body weight	Ao, PV, liver, kidney, spleen

Abbreviations: Ao = aorta; CVC = caudal vena cava; PV = portal vein.

**Table 4:** Summary details of the study included in the final systematic review evaluating contrast medium (CM)-related factors associated with enhancement in abdominal angio-CT in the dog.

First Author	Year	CM-Related Factor	Anatomical District
Tateishi K. [20]	2008	Injection technique	Ao, Liv
Kim C. [16]	2020	Injection technique	GI tract
Burgener F.A. [22]	1981	Injection technique	Aorta, CVC, Liv, Sp, Kid, Pan
Lee S.K. [14]	2017	Catheter size	Ao, PV, Liv
Kishimoto M. [11]	2008	CM formulation	Ao, CVC
Amaha T. [19]	2022	Injection technique	Ao, PV, Liv
Blaser A. [12]	2016	Injection technique	Adrenal glands
Choi S.Y. [21]	2018	Injection technique	Ao, PV, Liv, Pan
Chau J. [13]	2016	Injection technique	Ao, Liv
Davè A.C. [10]	2022	Injection technique	Ao, CVC, PV, Liv, Pan, Sp, Kid
Fitzgeral E. [15]	2017	Injection technique	GI tract
Choi S.Y. [25]	2016	Injection technique	Ao, PV, Liv, Pan
Kim H. [23]	2019	Saline chaser	Ao, PV, Liv
Thierry F. [9]	2018	Injection technique	Ao, PV, CVC

Abbreviations: Ao = aorta; CVC = caudal vena cava; Kid = kidney; GI = gastrointestinal; Liv = liver; Pan = pancreas; PV = portal vein; Sp = spleen.

**Table 5:** Summary details of the study included in the final systematic review evaluating scanner-related factors associated with enhancement in abdominal angio-CT in the dog.

First Author	Year	Scanner-Related Factor	Anatomical District
Choi S.Y. [24]	2015	Scan delay	Pancreas
Davè A.C. [10]	2022	Scan length and time	Ao, CVC, PV, Liv, Pan, Sp, Kid
Siow J.W. [27]	2023	Scan delay	GI tract
Choi S.Y. [25]	2016	Scan delay	Ao, PV, Liv, Pan
Cho H. [26]	2018	Scan delay	Kid
Thierry F. [9]	2018	Scanner type	Ao, PV, CVC

Abbreviations: Ao = aorta; CVC = caudal vena cava; Kid = kidney; GI = gastrointestinal; Liv = liver; Pan = pancreas; PV = portal vein; Sp = spleen.

## Data Availability

Newly generated data (reanalyzed from original work) are contained within the article.
